# Supplementary material for: Association between oral microbiome and seven types of cancers in East Asian population: a two-sample Mendelian randomization analysis
Source: Front Mol Biosci. 2023 Nov 21;10:1327893. doi: 10.3389/fmolb.2023.1327893 (PMC10702768; doi:10.3389/fmolb.2023.1327893)
Supplement: Supplementary file 1 [file DataSheet1.ZIP › Supplementary Materials/MR plots of saliva/saliva═╝/Lung Cancer/pheno.2213_to_lung cancer_forest.pdf]

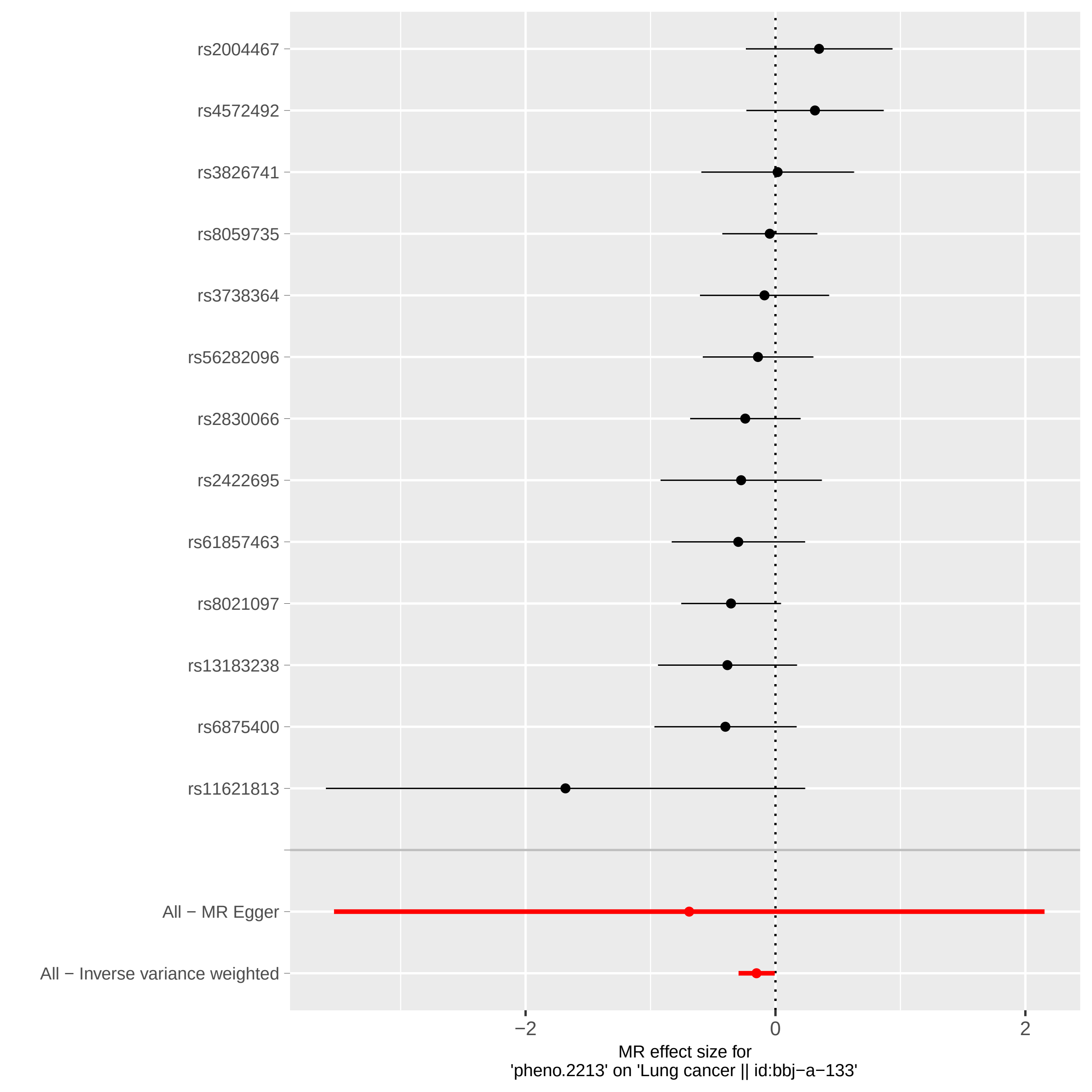

rs2004467
rs4572492
rs3826741
rs8059735
rs3738364
rs56282096
rs2830066
rs2422695
rs61857463
rs8021097
rs13183238
rs6875400
rs11621813
All − MR Egger
All − Inverse variance weighted
−2
0
2
MR effect size for
'pheno.2213' on 'Lung cancer || id:bbj−a−133'
